# Aberrant intra‐ and internetwork functional connectivity patterns of the anterior and posterior hippocampal networks in schizophrenia

**DOI:** 10.1111/cns.14171

**Published:** 2023-03-22

**Authors:** Xin‐Wei Li, Hong Liu, Yuan‐Yang Deng, Zhang‐Yong Li, Yu‐Hao Jiang, De‐Yu Li, Shu‐Yu Li

**Affiliations:** ^1^ School of Bioinformatics Chongqing University of Posts and Telecommunications Chongqing China; ^2^ School of Biological Science & Medical Engineering Beihang University Beijing China; ^3^ State Key Laboratory of Cognitive Neuroscience and Learning Beijing Normal University Beijing China

**Keywords:** hippocampal segments, internetwork, intranetwork, resting‐state functional connectivity, schizophrenia

## Abstract

**Aim:**

Schizophrenia is associated with abnormal hippocampal structure and function. Available evidence suggests that the anterior and posterior hippocampus are differentially affected by schizophrenia pathology. This study was designed to provide new insight into the anterior and posterior hippocampus in schizophrenia from the perspective of functional connectivity.

**Methods:**

Based on resting‐state functional magnetic resonance imaging data of 71 schizophrenia patients and 74 normal controls, we utilized a data‐driven approach to functionally segment the hippocampus into anterior and posterior segments and then investigated the functional connectivity patterns within and between the two hippocampal networks at the network, edge, and nodal levels.

**Results:**

We found that schizophrenia patients showed hyperconnectivity of both the anterior and posterior hippocampal networks. We also observed that the network alterations appear somewhat greater in the anterior hippocampal network than the posterior network, the left side than the right, and the intranetwork connectivity than the internetwork connectivity.

**Conclusion:**

The results reveal convergent and divergent intranetwork and internetwork connectivity patterns of the anterior and posterior hippocampus in schizophrenia, providing novel and important insights into the mechanisms of hippocampal pathology in schizophrenia.

## Graphical abstract

The anterior and posterior hippocampal networks in schizophrenia showed convergent and divergent intranetwork and internetwork connectivity patterns, at the network, edge, and nodal levels.

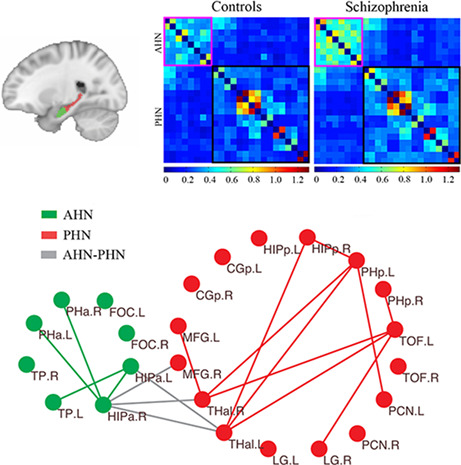

## 1 INTRODUCTION

Schizophrenia is a chronic and severe psychiatric disorder of unknown pathogenic mechanisms.^1^ Several lines of evidence suggest that the abnormalities of hippocampal structure and function are implicated in the pathophysiology of schizophrenia.^2^, ^3^, ^4^ The balance of excitation inhibition in the hippocampus is important for maintaining its normal function, and disruption of this balance may lead to the development of schizophrenia.^2^, ^5^ Existing research shows that impaired glutamatergic receptors and gamma‐aminobutyric acid (GABA)‐ergic neurons alter the microcircuit of the hippocampus and are thought to be a cause of its dysfunction.^2^, ^6^ Hippocampal dysfunction is suggested to be associated with cognitive deficits in schizophrenia and maybe a potential neuroimaging biomarker for diagnosing and treating.^7^, ^8^, ^9^ Furthermore, hippocampal hyperactivity derived from excitation‐inhibition imbalance is a robust and early endophenotype of schizophrenia.^7^, ^10^ This hyperactivity is hypothesized to be responsible for the hippocampal volume deficits in patients with schizophrenia.^2^, ^3^ In addition, the occurrence of schizophrenia is often accompanied by aberrant hippocampal connectivity.^11^, ^12^, ^13^

It is acknowledged that the hippocampus is heterogeneous and differs in structure and function along with its longitudinal axis,^14^, ^15^ usually accompanied by longitudinal changes of hippocampal subfield volumes.^16^, ^17^ In particular, the anterior and posterior portions of the hippocampus have different afferent and efferent projections to the rest of cortical and subcortical areas.^18^ Additionally, the anterior and posterior hippocampal networks are involved differently in verbal and visual memory.^19^ Moreover, the subfields are described as differentially affected in schizophrenia pathology, where the anterior hippocampus is more involved than the posterior.^3^, ^20^ Indeed, some structural imaging studies have revealed the reduced volume of the anterior but not the posterior hippocampus in schizophrenia.^21^, ^22^ On a functional level, Schobel et al. suggested that the anterior hippocampus (particularly the Cornu Ammonis 1 region) is an early and selective region affected in schizophrenia, and its hypermetabolism was tightly linked to the clinical symptoms of the disease.^23^, ^24^ Furthermore, a recent review paper proposed that the evolution of schizophrenia begins with the dysregulation of glutamate neurotransmission in the anterior hippocampal Cornu Ammonis 1 region and gradually expands to other brain regions.^3^ Specifically, it has been proposed that dysregulated neurotransmission of glutamatergic circuitry may lead to excitotoxic effects^25^ and abnormal hippocampal activation,^26^, ^27^ resulting in volumetric reductions.^28^

Considering schizophrenia may be caused by perturbation of the reciprocal cortico‐hippocampal pathways,^20^, ^29^, ^30^ it is significant to investigate the effects of schizophrenia on the connectivity pattern of the anterior and posterior hippocampus. Resting‐state functional MRI (R‐fMRI) provides a noninvasive technique for studying interregional functional connectivity in spontaneous brain activity.^31^, ^32^, ^33^ Concerning R‐fMRI studies in schizophrenia, Zhou et al.^11^ selected the anterior hippocampus as the region of interest and found its connectivity with other brain regions were reduced in schizophrenia compared to normal subjects. In a longitudinal R‐fMRI study, Kraguljac et al.^12^ reported a mixed pattern of increased and decreased connectivity with the anterior and posterior hippocampal seeds, and the baseline hippocampal connectivity can predict clinical improvement after treatment. Additionally, the anterior and posterior hippocampal networks showed a distinct relationship between their network modularity and relational memory.^30^ However, these studies mainly focused on the connectivity alterations within the anterior hippocampal network and/or the posterior hippocampal network, but not the interactions between the two hippocampal networks. Interactions between networks, reflecting information integration across spatially segregated brain regions, may help to increase the brain's resistance to pathological attacks.^34^, ^35^ Based on three levels of analysis (network level, node level, and edge level), several recent studies have revealed aberrant intra‐ and internetwork dysfunctions in various disorders, such as Alzheimer's disease, depression, and ischemic white matter lesions.^36^, ^37^, ^38^, ^39^ Thus, studying the organization of intra‐ and internetwork connectivity of the anterior and posterior hippocampal networks may improve our understanding of the pathophysiological mechanisms underlying schizophrenia.

Inspired by the above studies, we hypothesized the existence of convergent and divergent intranetwork and internetwork connectivity patterns of the anterior and posterior hippocampus in schizophrenia patients in comparison with normal controls. We also speculated that functional network properties might be associated with cognitive impairment severity in schizophrenia. To test these hypotheses, we first functionally segmented the hippocampus into anterior and posterior segments using a data‐driven approach.^40^ Then we evaluated the altered functional connectivity patterns within and between the two hippocampal networks at the network, edge, and nodal levels to further elucidate the role of the hippocampus in schizophrenia. Additionally, we investigated the relationships between the brain network measurements and cognitive performance by applying partial correlation analysis.

## 2 MATERIALS AND METHODS

### 2.1 Participants

The data used in this study were obtained from the Center for Biomedical Research Excellence (COBRE, fcon_1000.projects.nitrc.org/indi/retro/cobre.html) sample, which includes 72 schizophrenia patients and 74 normal controls (ages ranging from 18 to 65 in each group). Detailed study procedures can be found at https://www.cobre.mrn.org/. Briefly, all individuals were screened and excluded for a diagnosis of neurological illness, mental retardation, severe head trauma, substance abuse, or dependence within the last 12 months. Clinical diagnoses were assessed using the Structured Clinical Interview for DSM Disorders (SCID). Psychopathological symptoms were rated using the Positive and Negative Syndrome Scale (PANSS).^41^ The doses of antipsychotic medications at the MRI scan were converted to olanzapine equivalents.^42^ Intellectual functioning was assessed by the Wechsler Abbreviated Scale of Intelligence (WASI), which provides verbal IQ, performance IQ and full‐scale IQ (i.e., the average of verbal IQ and performance IQ). And cognitive symptoms were assessed by the MATRICS Consensus Cognitive Battery (MCCB) that measures seven cognitive domains including processing speed, attention/vigilance, working memory, verbal learning, visual learning, reasoning/problem solving, and social cognition.^43^ The MCCB also produces a composite score based on the scores for all domains. After the evaluation of head motion and image quality, one patient (ID: 40075) was excluded from further analysis. Table 1 shows a summary of the demographic and clinical information of the participants.

**TABLE 1:** Demographics and clinical characteristics of the participants.

	Schizophrenia (*n* = 71)	Controls (*n* = 74)	Test statistic (*p* value)
Age (years)	38.14 ± 13.99	35.82 ± 11.58	*z* = −0.799 (0.424)
Gender (male/female)	57/14	51/23	*χ* ^2^ = 2.462 (0.117)
Age of onsets (years)	22.03 ± 8.50	–	–
Illness duration (years)	16.51 ± 13.01	–	–
Medication OE dose	15.68 ± 35.19	–	–
PANSS			
Positive	14.85 ± 4.76	–	–
Negative	14.52 ± 4.86	–	–
General	29.15 ± 8.38	–	–
IQ	(*n* = 67)	(*n* = 67)	
Verbal	98.28 ± 16.50	106.69 ± 11.16	*t =* 3.454 (0.001)
Performance	102.81 ± 16.73	114.03 ± 12.32	*z =* −3.865 (<0.001)
Full scale	99.91 ± 16.77	111.66 ± 11.83	*z* = −4.062 (<0.001)
MCCB	(*n* = 67)	(*n* = 67)	
Processing speed	35.91 ± 12.02	54.13 ± 8.73	*t =* 10.042 (<0.001)
Attention/vigilance	36.80 ± 13.72	49.03 ± 8.80	*t =* 6.037 (< 0.001)
Working memory	40.02 ± 12.47	50.40 ± 10.54	*z =* −4.725 (<0.001)
Verbal learning	38.78 ± 8.48	46.03 ± 8.84	*z =* −4.472 (<0.001)
Visual learning	36.87 ± 11.83	45.36 ± 10.05	*t =* 4.478 (<0.001)
Reasoning/problem solving	43.92 ± 10.75	55.31 ± 10.81	*z =* −5.677 (<0.001)
Social cognition	41.21 ± 12.01	51.78 ± 9.83	*t =* 5.575 (<0.001)
Overall composite score	32.20 ± 13.24	50.07 ± 8.22	*z =* −7.366 (<0.001)

*Note*: Data are presented as mean ± standard deviation. Chi‐squared test was used for gender comparisons; Two‐sample tests were used for normally distributed continuous variables; Mann–Whitney *U*‐tests were used for non‐normally distributed continuous variables.

Abbreviations: MCCB, MATRICS Consensus Cognitive Battery; OE, olanzapine equivalents; PANSS, positive and negative syndrome scale.

All procedures performed were in accordance with the ethical standards of the institutional and/or national research committee and with the 1964 Helsinki declaration and its later amendments or comparable ethical standards. Informed consent was obtained from all individual participants included in the study.

### 2.2 Image acquisition

For each subject, a structural MRI and an R‐fMRI were scanned using a Siemens Tim‐Trio 3T scanner. Structural images were acquired via a multiecho MPRAGE sequence with the following parameters: field‐of‐view (FOV) = 256 × 256 mm^2^, matrix size = 256 × 256 × 176, voxel size = 1 × 1 × 1 mm^3^, flip angle = 7°, number of echoes = 5, TR/TE/TI = 2530/[1.64, 3.5, 5.36, 7.22, 9.08]/900 ms, and scan duration = 6:03 min. Functional images were acquired using an echo‐planar imaging sequence with the following parameters: matrix size = 64 × 64, 33 slices, voxel size = 3.8 × 3.8 × 3.5 mm^3^, TR/TE = 2000/29 ms, 150 volumes, and scan duration = 5:04 min. The R‐fMRI scans were done with eyes open.

### 2.3 Image preprocessing

Image preprocessing was conducted in FMRIB's Software Library v5.0 (fsl, https://www.fmrib.ox.ac.uk/fsl). Structural image preprocessing steps included bias field correction, registered to MNI standard space using linear and nonlinear registration, brain extraction and tissue‐type segmentation into gray matter, white matter, and cerebrospinal fluid. Functional image preprocessing steps included discarding the first six volumes, motion correction by registering each volume in a time series to the middle volume, slice‐timing correction, brain extraction, spatial smoothing with a 5‐mm FWHM Gaussian kernel, temporal band‐pass filtering (0.01–0.08 Hz), alignment to the individual structural space using boundary‐based registration method,^44^ and regressed out several nuisance signals (6 head motion parameters, major Eigen signals from white matter, cerebrospinal fluid, and global brain). The major Eigen time series is a single time series that best reflects the mask's activity by representing the maximum variance within the mask,^45^ which can be extracted by the fslmeants function provided in fsl software. It is less susceptible to registration errors than the mean time series, thus helping to avoid the mixing of signal and noise.^45^

### 2.4 Functional divisions of the hippocampus

**FIGURE 1:**
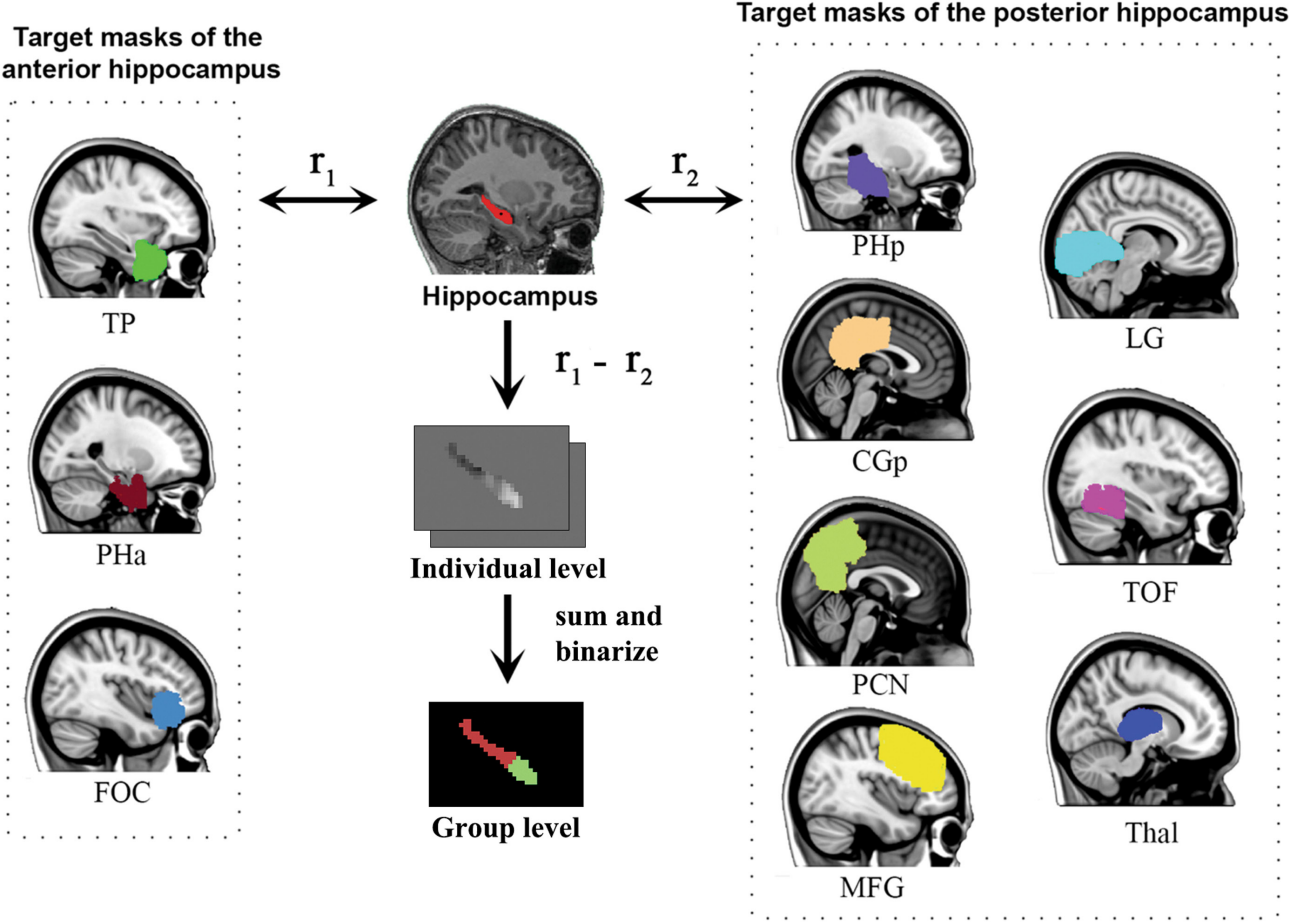
Illustration of functional divisions of the hippocampus. The *r*_1_ and *r*_2_ represent the partial correlation coefficients between the BOLD time series of one hippocampal voxel and the major Eigen time series of the anterior hippocampal target masks, and the posterior hippocampal target masks, respectively. If *r*_1_ > *r*_2_, the voxel is classified into the anterior hippocampus, otherwise the posterior hippocampus. A subtraction map is obtained by *r*_1_ minus *r*_2_ for each individual. The individual subtraction maps of a group are then summed and binarized to obtain the group‐level hippocampal segmentation results. TP, temporal pole; PHa, parahippocampal gyrus, anterior division; FOC, frontal orbital cortex; PHp, parahippocampal gyrus, posterior division; CGp, cingulate gyrus, posterior division; PCN, precuneous cortex; MFG, middle frontal gyrus; LG, lingual gyrus; TOF, temporal occipital fusiform cortex; Thal, left/right thalamus.

The seed‐based analysis like Kraguljac et al.^12^ relies on subjective ROIs selection, which can affect the way we interpret the data.^46^ Besides, ROIs based on anatomical landmarks or cytoarchitectonic boundaries contain little information about connectivity, limiting their ability to accurately represent connectomes.^47^, ^48^ Moreover, evidence suggests that the anatomical borders of the anterior and posterior hippocampus may not correspond well with their functional divisions.^49^ Therefore, following the relevant studies on segmenting the hippocampus functionally,^50^, ^51^ we adopted a data‐driven approach to identify functional divisions within the hippocampus unbiased by subjective anatomical delineations or group status for subsequent connectome analysis (as shown in Figure 1). This approach subdivides the original anatomically defined hippocampus into regions with homogeneous connectivity (supposed of homogeneous function), which can refine the used brain anatomical atlas (i.e., the Harvard‐Oxford atlas), indirectly enhancing the investigation of macroscopic connectome features.^46^

First, the target masks of the anterior and posterior hippocampus were selected based on their demonstrated functional connections.^52^, ^53^, ^54^ For the anterior hippocampus, three brain regions labeled “Temporal pole,” “Parahippocampal Gyrus, anterior division,” and “Frontal Orbital Cortex” were combined (i.e., adding binary images of separate ROIs together) to create a target mask related to the anterior hippocampus. And seven brain regions labeled “Parahippocampal Gyrus, posterior division,” “Cingulate Gyrus, posterior division,” “Precuneous Cortex,” “Middle Frontal Gyrus,” “Lingual Gyrus,” “Temporal Occipital Fusiform Cortex,” and “Left/Right Thalamus” were combined to create a target mask related to the posterior hippocampus. All anatomical brain regions were derived from the Harvard‐Oxford cortical and subcortical structural atlases provided in fsl software. The hippocampus and two combined target masks were registered to the individual structural space and confined to the gray matter mask based on the tissue‐type segmentation and then transformed to the individual functional space. The intensity threshold of the gray matter mask was set at 0.2. The registration file used here was the inverse of the transformation obtained in the image preprocessing step.

**FIGURE 2:**
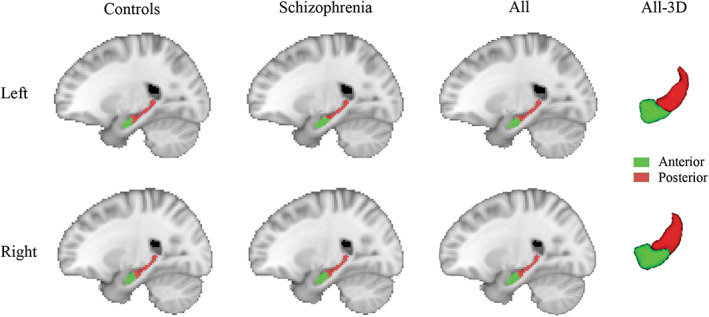
Hippocampal functional divisions. Anterior and posterior hippocampus in normal controls and schizophrenia patients are shown in the sagittal view. Group‐unbiased divisions from all subjects are shown in sagittal view and 3D view.

For each target mask, the major Eigen time series was extracted. Then for each voxel in the hippocampus, we calculated the partial correlation scores (i.e., *r*_1_ and *r*_2_) of its BOLD time series with one target mask's major Eigen time series, while controlling for potential effects of the other target mask's major Eigen time series. This yielded two correlation maps of the hippocampus corresponding to the anterior and posterior target masks, respectively. We then transformed the two correlation maps into standard MNI space and subtracted the correlation map of the posterior mask from the correlation map of the anterior mask to obtain an individual subtraction map (i.e., *r*_1_ − *r*_2_), where the positive value indicates that the voxel is more related to the anterior hippocampus, while the negative value belongs to the posterior hippocampus. The individual subtraction maps were then summed for controls and schizophrenia, respectively, and then binarized (threshold = 0) to produce group‐level hippocampal divisions (Figure 2). The group‐unbiased hippocampal division was also generated for subsequent analysis by summing the subtraction maps of all subjects and binarization.

### 2.5 Network construction

The generated anterior hippocampus and corresponding three target masks (hemisphere‐specific) were treated as nodes of the anterior hippocampus network, and the posterior hippocampus and seven target masks were treated as nodes of the posterior hippocampus network. These regions were confined to the gray matter mask and registered to the native functional space. Pearson's correlation coefficients (*r*) of the major Eigen time series between any pair of regions were calculated and then converted to *z*‐scores using Fisher's *r*‐to‐*z* transformation, forming a 24 × 24 undirected weighted matrix per subject. After setting the negative edges to zero, group‐level *z*‐transformed connectivity matrices were computed by averaging the individual matrices for controls and schizophrenia, respectively. Here, we remove the negative correlations for the following reasons. The meaning of negative correlations is currently unclear. Studies have suggested that negative correlations may arise from preprocessing steps, particularly global signal regression, rather than from actual inhibitory interactions between brain regions.^55^, ^56^ Furthermore, the positive and negative correlations exhibit different organizations^57^ and should be considered separately when calculating the mean functional connectivity for a group, otherwise, they would cancel out each other. Also, most of network measures are not suitable for the brain networks that contain both positive and negative correlations.^58^, ^59^ In addition, negative correlations may lead to low test–retest reliability.^60^ Given these problems, it is common practice to remove them in brain network analyses.^61^, ^62^

### 2.6 Functional network analysis

Inspired by previous studies,^36^, ^39^ we analyzed the intra‐ and internetwork connections of the hippocampus‐related networks in controls and schizophrenia the following three levels:

(1) Network level: The intra‐ and internetwork connectivity strength was investigated as previously described.^63^ The composite score ($C_{p}$) that indicates the intranetwork connectivity strength is defined as

$$C_{p}=\frac{1}{N_{p}\times \left(N_{p}-1\right)}\sum_{i,j\in p,i\neq j}z_{\mathrm{ij}}$$

where $N_{p}$ is the number of nodes within the network *p*, and $z_{\mathrm{ij}}$ represents the *z*‐transformed correlation value between two nodes within network *p*. Similarly, the composite score ($C_{p,q}$) that indicates the internetwork connectivity strength is defined as

$$\begin{array}{c} C_{p,q}=\frac{1}{N_{p}\times N_{q}}\sum_{i\in p,j\in q}z_{\mathrm{ij}} \end{array}$$

where $N_{p}$ and $N_{q}$ are the number of nodes within the network *p* and *q*, respectively, and $z_{\mathrm{ij}}$ represents the *z*‐transformed correlation value between node *i* of network *p* and node *j* of network *q*.

(2) Edge level: Measurements at the network level promote data reduction and reduce sampling errors in statistical analysis, but may obscure focal abnormalities.^63^ Thus, we also investigated the group differences in functional connectivity, i.e., the correlation value $z_{\mathrm{ij}}$ between all node pairs.

(3) Nodal level: For each node *i*, its integration was computed as $k_{i}=\sum_{j\in N,i\neq j}z_{\mathrm{ij}}$, where $z_{\mathrm{ij}}$ represents the *z*‐transformed correlation value between node *i* and all other nodes in the network, and *N* refers to the number of nodes. The nodal integration describes how well a node is connected in the network, which is equivalent to the weighted degree of the node in the graph theory.

### 2.7 Statistical analysis

Statistical analyses were conducted using Statistical Package for Social Sciences (SPSS, version 23.0) and MATLAB (2018a; Mathworks). The normality of continuous variables was assessed using Kolmogorov–Smirnov tests. All statistical tests were two‐tailed. For the demographics and cognitive scores, two‐sample *t*‐tests (for normally distributed continuous variables), Mann–Whitney *U*‐tests (for non‐normally distributed continuous variables), and a Chi‐squared test (for gender) were used to investigate the differences between controls and schizophrenia groups. We also compared the volume of the anterior hippocampus and posterior hippocampus between groups using the two‐sample *t‐*test. The significance threshold was set at *p <*0.05.

To assess the differences in network measures between controls and schizophrenia, we used generalized linear models suitable for response variables with arbitrary distributions, where age and gender as covariates of no interest. For network‐level measures (i.e., intra‐/internetwork connectivity), a Bonferroni‐corrected significant threshold was set at *p* <0.0167 (0.05/3, number of tests = 3). In the statistical comparisons of functional connectivity, the group difference map was first threshold at *p* < 0.01, followed by multiple comparisons correction using the network‐based statistics (NBS) method (number of tests/connectivities = 276). The NBS is an effective nonparametric statistical method for controlling the family‐wise error rate and has been widely used to identify connections associated with a between‐group difference.^64^ The permutation tests were repeated 1000 times, and the significance threshold was set at $p_{\text{corrected}}<0.05$. Furthermore, for the nodal degree, a two‐tailed *p* < 0.05 was considered statistically significant using a false discovery rate (FDR) correction method for multiple comparisons (number of tests/nodes = 24). Here, different correction methods were selected based on the type and number of tests.

Besides, to evaluate the relationships between the altered network measures at the three levels and MCCB cognitive scores, partial correlation analyses were performed after controlling for age and gender. To ensure that the correlations between cognitive scores and network measures are not misleading due to group difference effects, we first investigated their relationships in each group separately. If both groups reported significant correlations, further investigation was performed among all subjects. We also evaluate the correlations between aberrant network measures and patients' symptoms, including PANSS positive, negative, and general scores. A threshold level of *p* < 0.05 (FDR corrected) was considered significant.

## 3 RESULTS

### 3.1 Demographic and clinical data

Table 1 shows the demographic and cognitive data of the controls and patients with schizophrenia. There were no significant differences in age (*p* = 0.424) or gender (*p* = 0.117) between groups. Controls had significantly higher verbal, performance and full‐scale IQ than schizophrenia patients (*p* ≤ 0.001). However, we did not enter IQ as a covariate in the network analysis because pervasive cognitive impairments are characteristics of schizophrenia,^65^ so controlling for its effects in the statistical analysis may be inappropriate so it may not be appropriate to control its effects in the statistical analysis.^66^, ^67^ And the MCCB scores in all cognitive domains and the overall composite scores of the schizophrenia group were significantly lower than that in the control group (*p* < 0.001).

### 3.2 Functional divisions of the hippocampus

Using the data‐driven approach, we obtained the functional divisions of the hippocampus in controls and schizophrenia groups (Figure 2). Highly consistent with prior findings,^50^, ^51^ the approximate anterior 1/3 portion along the hippocampal longitudinal axis shows preferential correlation with the anterior cortical regions, and the posterior 2/3 portion is more correlated with the posterior cortical regions. As visualized, the anterior–posterior functional divisions were globally matched between groups. Statistical analysis also confirmed no significant group differences in anterior and posterior hippocampal volume between schizophrenia and control (*p* > 0.05). Thus, we used the group‐unbiased divisions from all subjects in the following network analysis.

### 3.3 Network‐level analysis

**FIGURE 3:**
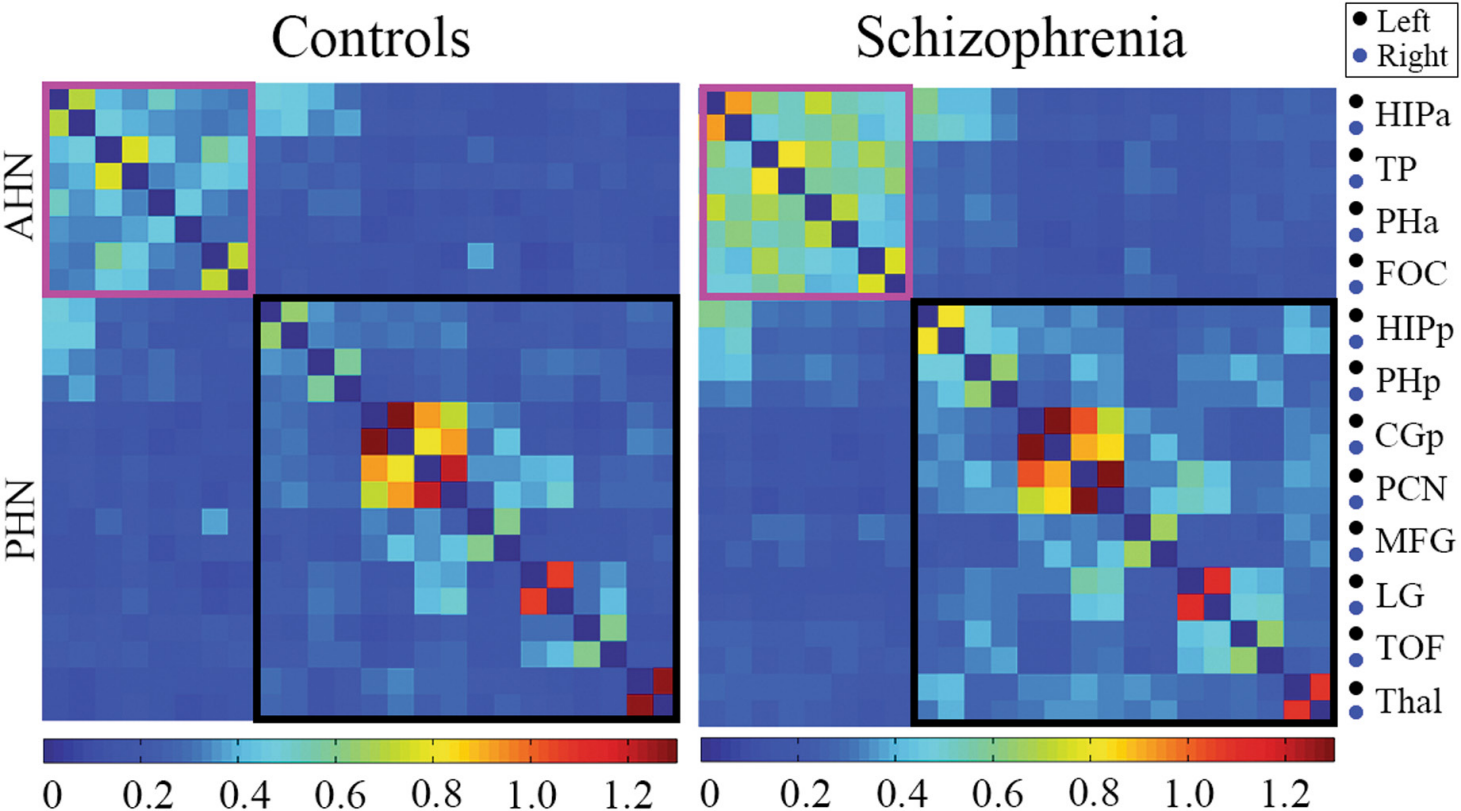
Mean *z*‐transformed connectivity matrices in control and schizophrenia groups. Nodes are grouped according to network membership. Intranetwork correlations appear in diagonal blocks, and internetwork correlations appear in off‐diagonal blocks. AHN, anterior hippocampal network; PHN, posterior hippocampal network; HIPa, anterior hippocampus; TP, temporal pole; PHa, anterior parahippocampal gyrus; FOC, frontal orbital cortex; HIPp, posterior hippocampus; PHp, posterior parahippocampal gyrus; CGp, posterior cingulate gyrus; PCN, precuneous cortex; MFG, middle frontal gryus; LG, lingual gyrus; TOF, temporal occipital fusiform cortex; Thal, thalamus.

**FIGURE 4:**
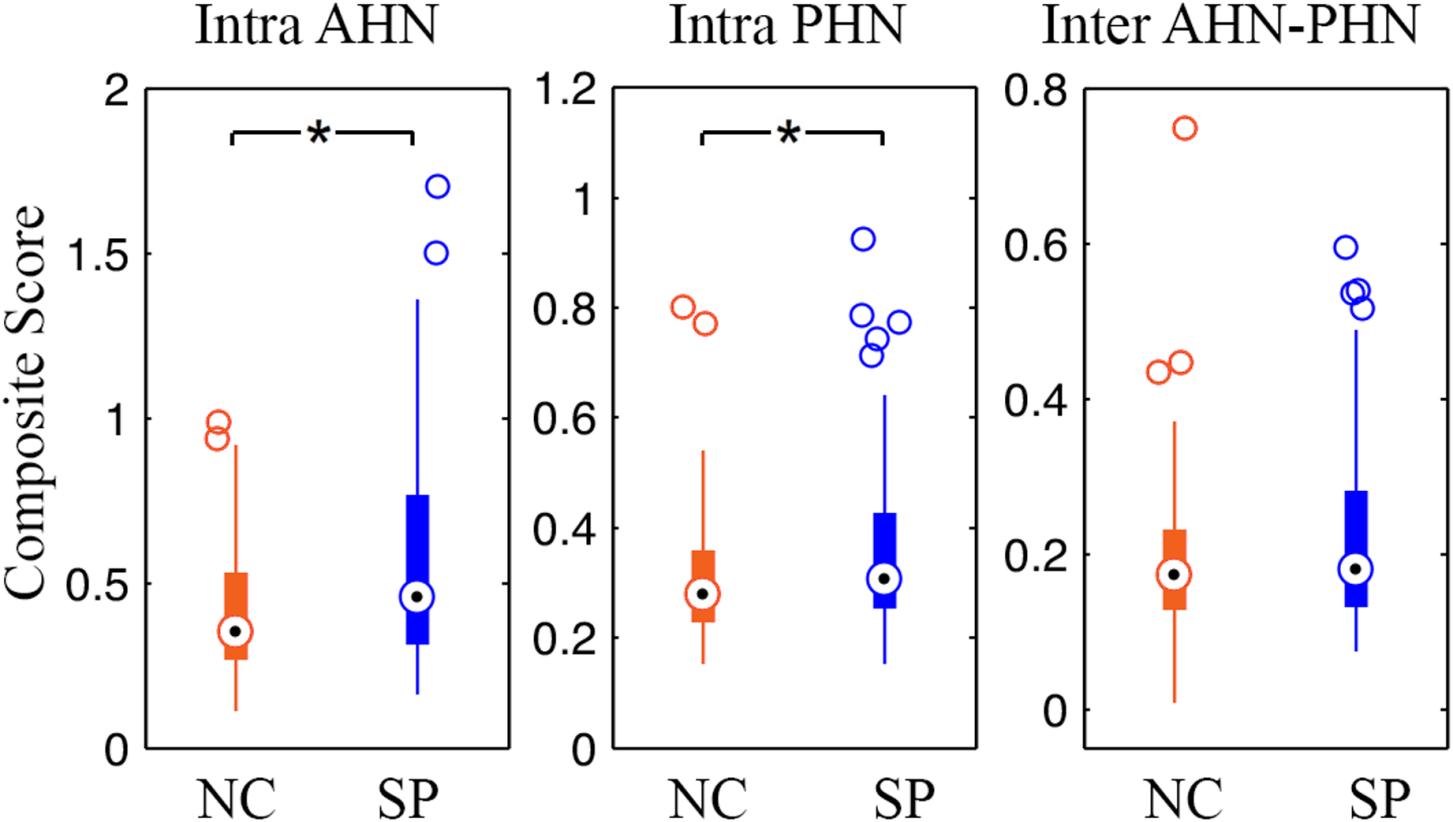
Between‐group differences in intranetwork and internetwork connectivity. Asterisks denote significant between‐group differences (*p <* 0.05, Bonferroni corrected). AHN, anterior hippocampal network; PHN, posterior hippocampal network; NC, normal controls; SP, schizophrenia.

We generated the mean *z*‐transformed connectivity matrices for control and schizophrenia groups (Figure 3). The intranetwork correlations within the anterior and posterior hippocampal networks appear in diagonal blocks, and the internetwork correlations between them are shown in off‐diagonal blocks. We can observe that the connections within each network are stronger than cross‐networks, especially in the schizophrenia group. Group comparisons of the intra‐ and internetwork composite scores are shown in Figure 4. Significantly increased intranetwork composite scores in the schizophrenia patients compared with controls were found in both anterior (*p* = 0.002) and posterior hippocampal networks (*p* = 0.008). In contrast, no significant group difference was found the internetwork composite scores. Moreover, we found no significant correlations between network measures at three levels and PANSS symptom scores or MCCB cognitive scores in the schizophrenia group.

### 3.4 Edge‐level analysis

**FIGURE 5:**
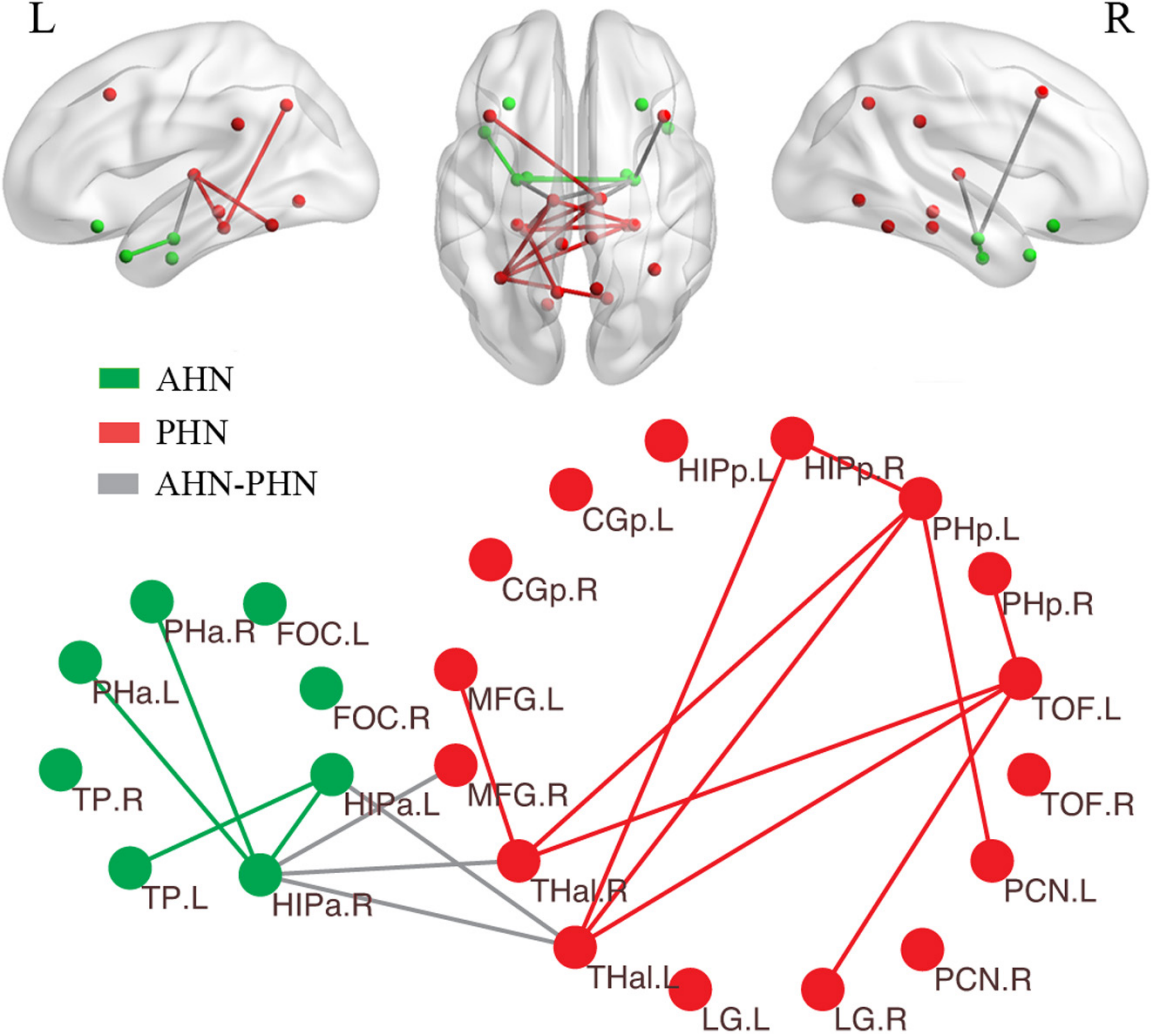
Altered functional connectivity in schizophrenia. The 3D distribution (upper row) and 2D representations (lower row) of the edges with significant between‐group differences at *p <* 0.05 (NBS corrected) are shown, among which the green lines indicate the AHN intranetwork edges, red lines indicate the PHN intranetwork edges, and gray lines indicate the internetwork edges. AHN, anterior hippocampal network; PHN, posterior hippocampal network; HIPa, anterior hippocampus; TP, temporal pole; PHa, anterior parahippocampal gyrus; FOC, frontal orbital cortex; HIPp, posterior hippocampus; PHp, posterior parahippocampal gyrus; TOF, temporal occipital fusiform cortex; Thal, thalamus; LG, lingual gyrus; PCN, precuneous cortex; CGp, posterior cingulate gyrus; MFG, middle frontal gryus; L, left; R, right.

We further investigated the altered functional connectivity across node pairs. Group comparisons revealed that 18 out of 276 edges (*p* < 0.05, NBS corrected) showed significantly increased correlations in the schizophrenia group compared with controls (Figure 5). Among them, four edges were categorized as internetwork connections (4/128, 3.1%) and 14 edges were categorized as intranetwork connections, including four edges within the anterior hippocampal network (4/28, 14.3%) and ten edges within the posterior hippocampal network (10/120, 8.3%). Moreover, the regions with the most abnormal connections were anterior hippocampus and thalamus (9 edges for each). Specifically, all altered edges within the anterior hippocampal network were connected with anterior hippocampus (4/4, 100%), six out of ten altered edges within the posterior hippocampal network were connected with thalamus (6/10, 60%), and three altered internetwork edges were connected between anterior hippocampus and thalamus (3/4, 75%). In Figure 5, the 3D distribution of the altered edges is visualized by the BrainNet Viewer,^68^ and the 2D representation is visualized by Pajek software.^69^

### 3.5 Nodal‐level analysis

**FIGURE 6:**
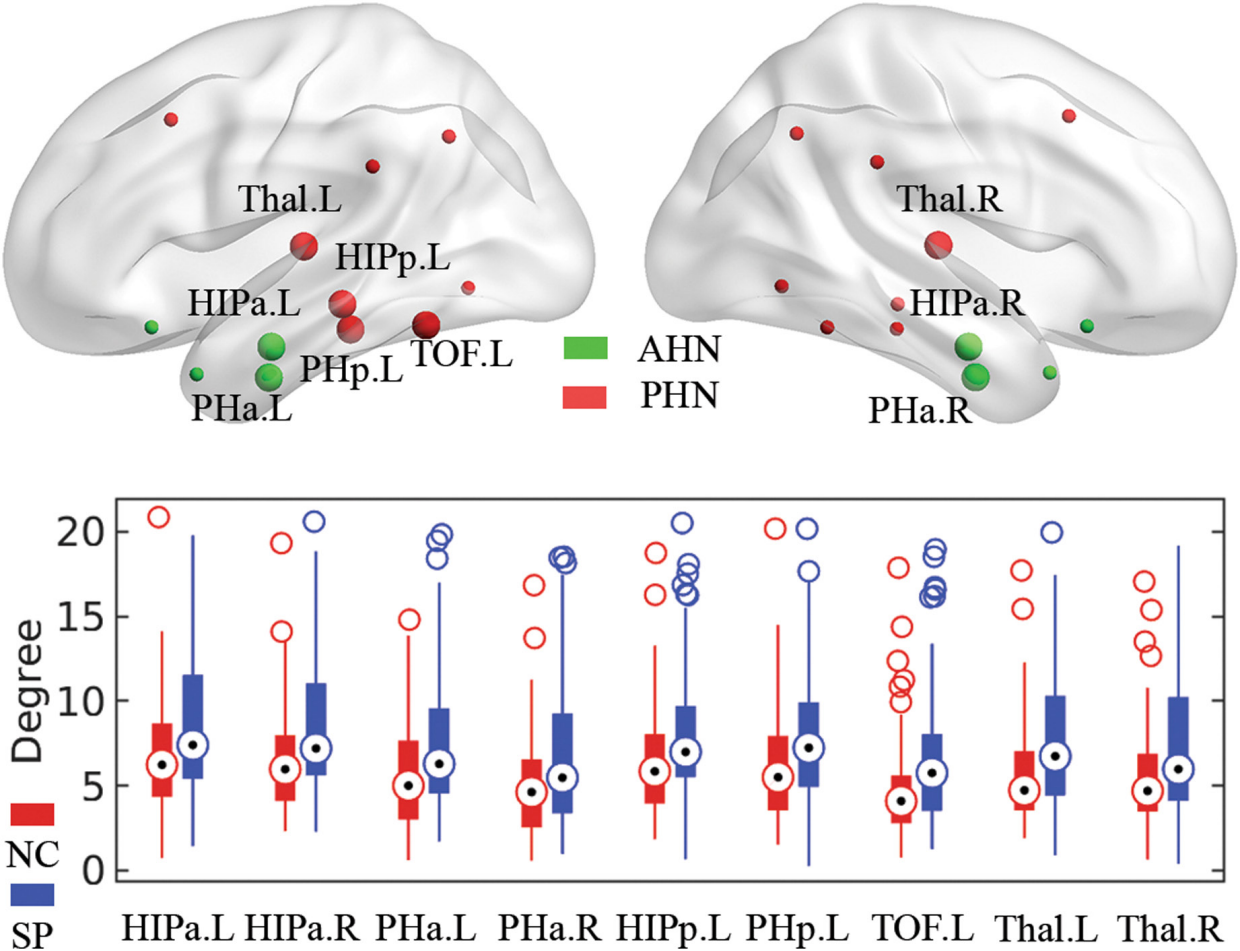
Altered nodal degree in schizophrenia. The upper row shows the 3D distribution of brain regions. The larger spheres with labels represent the nodes with significant between‐group differences at *p <* 0.05 (FDR corrected), the green spheres indicate the nodes of AHN, and the red spheres indicate the nodes of PHN. The lower row shows the boxplots of the nodal degree in controls and schizophrenia groups for those significant nodes. AHN, anterior hippocampal network; PHN, posterior hippocampal network; NC, normal controls; SP, schizophrenia; HIPa, anterior hippocampus; HIPp, posterior hippocampus; PHa, anterior parahippocampal gyrus; PHp, posterior parahippocampal gyrus; TOF, temporal occipital fusiform cortex; Thal, thalamus; L, left; R, right.

Next, we explored the between‐group differences in nodal degree. The 3D locations of the aberrant nodes (*p <* 0.05, FDR corrected) are highlighted as larger spheres in the upper row of Figure 6, and the boxplots in the lower row provide additional statistical information. Compared with the controls, 9 out of 24 nodes with an increased degree were identified in the schizophrenia group, including bilateral anterior hippocampus, anterior parahippocampus and thalamus, left posterior hippocampus, posterior parahippocampus, and temporal occipital fusiform cortex. Among them, four regions belong to the anterior hippocampal network (4/8, 50%) and the other five regions belong to the posterior hippocampal network (5/16, 31.3%). Noted that six out of nine aberrant nodes (66.7%) are located in the left hemisphere.

## 4 DISCUSSION

In this study, we investigated the aberrant intra‐ and internetwork connectivity patterns of functionally defined anterior/posterior hippocampus from three levels (network, edge, and node) in schizophrenia subjects. The results suggest that the abnormal changes in schizophrenia appear somewhat greater in the anterior hippocampal network than the posterior network, the left side than the right, and the intranetwork connectivity than the internetwork connectivity.

We observed hyperconnectivity of hippocampal networks at rest in schizophrenia patients compared with normal controls based on three‐level analysis, which is consistent with previous studies.^7^, ^70^ Such results may be related to dysfunction of glutamatergic receptors (primarily N‐methyl‐D‐aspartate receptors, NMDAR) and GABAergic inhibitory interneurons in schizophrenia.^6^, ^71^ The importance of the hippocampus in the NMDAR/GABA mechanisms of schizophrenia has been well described.^2^, ^20^, ^72^ Increased whole‐brain global resting‐state functional connectivity was found after the administration of NMDA receptor antagonist ketamine in healthy subjects, and this hyperconnectivity was associated with schizophrenia symptoms.^73^ Besides, Grimm et al.^74^ used the R‐fMRI data collected 20 min after ketamine injection to find an increase in the prefrontal‐hippocampal connection. However, a recent study reported decreased hippocampal connectivity during ketamine administration.^75^ This inconsistency may be due to the different temporal window of data collection and further research is needed. Moreover, previous studies combining magnetic resonance spectroscopy and R‐fMRI suggest that local GABA level correlated negatively with network connectivity.^76^, ^77^ However, inconsistent with our finding of increased hippocampal connectivity in schizophrenia, decreased,^11^, ^78^ mixed increased and decreased,^12^, ^79^ or normal^80^ hippocampal connectivity was also observed. The discrepancies might be partially due to different connectivity approaches or ROI selections.

Our results indicated that the anterior hippocampal network was more affected than the posterior based on the edge and nodal‐level analysis. This is somewhat consistent with previous findings that the anterior hippocampal network shows greater dysconnectivity than the posterior hippocampal network in patients with schizophrenia, schizoaffective disorder, and psychotic bipolar I disorder.^81^ Blessing et al.^82^ also found that anterior, but not posterior, hippocampal‐cortical functional connectivity can discriminate naïve first‐episode psychosis patients from controls. In addition, several neuroimaging studies have revealed that the anterior hippocampus is more implicated in schizophrenia than the posterior division regarding smaller volume,^21^, ^22^, ^83^ greater shape deformity,^84^, ^85^, ^86^ and hypermetabolism.^23^, ^24^ Furthermore, a previous animal study demonstrated dysfunction of the ventral hippocampus (corresponding to anterior in humans) leads to dopamine dysregulation in schizophrenia, which underlies the psychotic symptoms observed in this disorder.^87^, ^88^ Evidence also suggests schizophrenia may start from the anterior hippocampus and subsequently spread to other brain regions.^3^, ^23^ The findings here provide further support for the long‐axis specialization of the hippocampus in schizophrenia.

Moreover, our findings suggest that the heightened hippocampal connection in schizophrenia seem to more lateralized toward the left side, whereas 75% of aberrant nodes are located in the left hemisphere. In line with this finding, previous studies have revealed abnormal volumetric and morphological asymmetries of the hippocampus in patients with schizophrenia.^84^, ^89^, ^90^, ^91^ Studies of glutamate receptors also suggest that the left hippocampus was more affected than the right one in this disease.^92^ Furthermore, a meta‐analysis of tractography studies of schizophrenia reported that altered structural connectivity of the hippocampus was limited to the left hemisphere.^13^ The lateralization may be associated with the naturally smaller volume of the left hippocampus,^93^ which makes it more vulnerable to schizophrenia pathology than the right side.

Another finding is that the altered connectivities are mainly located within the anterior and posterior hippocampal networks, but rarely between the two networks. Dysconnectivity of both the two hippocampal networks in schizophrenia was also reported in a recent study using a priori defined hippocampal seeds.^12^ From our edge and nodal analysis, we can see the anterior hippocampus is the most affected region in the anterior hippocampal network, which is uniform with its important role in schizophrenia as discussed above. However, the most impaired region in the posterior hippocampal network is the thalamus. This finding may suggest a possible relationship between the hippocampal dysfunction model and thalamic dysfunction model^94^ of schizophrenia, which needs to be further investigated. In addition, the functional and neurobiological differences between the anterior and posterior hippocampus suggest that they have specialized roles in cognitive processing. Previous animal studies have shown that there is almost no direct fiber connectivity between the anterior and posterior hippocampus.^49^ And the anterior and posterior hippocampus have different connections to the cerebral cortex.^52^, ^53^, ^54^ The anterior hippocampal network is thought to be primarily involved in verbal memory, whereas the posterior hippocampal network plays an important role in nonverbal memory.^19^, ^54^ The preservation of internetwork connectivity found in this study may further support that each hippocampal network is specialized for specific functions, and the interaction between these two networks is not vulnerable to schizophrenia pathology.

Our findings highlight that abnormalities in the functional connectivity of hippocampal subregions are an important feature of schizophrenia which may, however, not significantly associated with schizophrenia symptoms or cognitive task performance. Consistent with our findings, a previous study of hippocampal resting state functional connectivity found no significant relationship between the hippocampal connectivity and symptom or cognitive variables in schizophrenia.^95^ This finding was repeated in an investigation of patients with primary DSM psychosis diagnoses.^81^ With regard to hippocampal structure, a recent review reported that the associations between hippocampal subregional volumes and symptom severity and cognitive performance tend to be weak.^96^ However, there is currently inconsistent evidence regarding the relationships between hippocampal metrics and symptom and cognitive scores in schizophrenia. For example, Kraguljac et al.^12^ reported that anterior and posterior hippocampal connectivity patterns were associated with negative symptom severity in unmedicated patients with schizophrenia. Moreover, Dugré et al.^97^ showed that hippocampal subregional connectivity can predict memory accuracy during episodic tasks. Robust findings may benefit from meta‐ or mega‐analyses in the future.

Several issues related to this study should be noted. First, since the schizophrenia is a heterogeneous disorder, it would be interesting to investigate the hippocampal networks in schizophrenia subtypes in our future research. Second, the current data set is cross sectional, therefore not allowing us to observe hippocampal intranetwork and internetwork changes with schizophrenia progression. Hence, future studies could be conducted to examine longitudinal schizophrenia‐related changes in the anterior and posterior hippocampal networks. Third, the detail of hippocampal connection patterns may be limited by the voxel resolution of the functional images we used. In the future, an optimized imaging sequence can be used to obtain more detail and accurate hippocampal dysconnectivity patterns in patients with schizophrenia. Fourth, we have ignored the negative functional correlations in our experiments for the reasons mentioned in the method. However, negative correlations do exist in spontaneous brain activity. It will be interesting to further investigate the anticorrelated brain networks of the anterior and posterior hippocampus in patients with schizophrenia in the future. Finally, the scan duration of the rsfMRI in the COBRE data set is 5:04 min. It should be noted that a previous study has reported that the reliability of functional connectivity can be greatly improved as the scan duration of rsfMRI increases from 5 min to 13 min.^98^ In the future, we woulf like to investigate the effect of scan length on our results. However, we consider our current results to be acceptable, as previous studies have shown that a scan duration of 5–7 min can result in stable estimates.^99^, ^100^

## 5 CONCLUSION

In summary, we have demonstrated that in patients with schizophrenia, there are convergent and divergent altered patterns in the intra‐ and internetwork connections of both the anterior and posterior hippocampal networks at rest. The results of this study reveal abnormal heightened connections in the hippocampal networks in schizophrenia compared with controls. Based on the three‐level analysis, the results further suggest that the hippocampal dysconnectivity pattern show divergence along the hippocampal long‐axis, between the two hemispheres and between the intra‐ and internetwork connections. These findings help to advance our understanding of the mechanisms of hippocampal pathology in schizophrenia.

## AUTHOR CONTRIBUTIONS

Xin‐Wei Li, Shu‐Yu Li, and De‐Yu Li designed the study. Xin‐Wei Li analyzed the data and wrote the first draft of the manuscript. Hong Liu and Yuan‐Yang Deng undertook the statistical analyses. Zhang‐Yong Li and Yu‐Hao Jiang analyzed the results. All authors contributed to and have approved the final manuscript.

## CONFLICT OF INTEREST STATEMENT

The authors declare no conflict of interest.

## Data Availability

All MRI data used in this study are publicly available at the Center for Biomedical Research Excellence (COBRE, fcon_1000.projects.nitrc.org/indi/retro/cobre.html).
